# Solid-State Metalloproteins—An Alternative to Immobilisation

**DOI:** 10.3390/molecules21070919

**Published:** 2016-07-14

**Authors:** Trevor D. Rapson

**Affiliations:** Commonwealth Scientific and Industrial Research Organisation (CSIRO), Black Mountain, Canberra, ACT 2601, Australia; trevor.rapson@csiro.au

**Keywords:** biocatalysis, industrial biotechnology, silk, biosensors, de novo engineering

## Abstract

This commentary outlines a protein engineering approach as an alternative to immobilisation developed in our laboratory. We use a recombinant silk protein into which metal active sites can be incorporated to produce solid-state metalloprotein materials. The silk protein directly coordinates to the metal centres providing control over their reactivity akin to that seen in naturally occurring metalloproteins. These solid-state materials are remarkably stable at a range of temperatures and different solvent conditions. I discuss the genesis of this approach and highlight areas where such solid-state materials could find application.

Enzymes hold great promise to provide green alternatives from traditional industrial processes. However, in order for an enzyme’s full potential to be realised, their use needs to be economically viable. Immobilisation of enzymes is a promising method to reduce the cost of production and processing, as it allows multiple uses of a single batch of enzymes and ease of product separation. In addition, immobilisation can also lead to an improvement of other enzymatic properties such as stability, selectivity, and specificity [1]. While achieving immobilisation without compromising the functional properties of enzymes is extremely challenging, great progress has been made in this research area [2,3]. Some examples include the use of magnetic nanoparticles [4], coupling purification and immobilisation into a single step [5], and developing a detailed understanding of the ideal physical properties of the solid support [6].

In recent years my research has focused on developing an alternative approach to enzyme immobilisation with the goal of developing extremely robust enzymes for industrial applications. Our strategy has been to use a silk protein as an engineering scaffold into which metal cofactors can be incorporated to introduce enzymatic function into the silk materials. Hence giving us solid state metalloprotein materials.

In the natural world, silks are almost always fibres. Silk proteins however can be fabricated in other material formats such as sponges and films. It was these unusual films that initially got me interested in silks (Figure 1A).

I was a postdoc developing nitric oxide sensors and looking for a new way to immobilise my nitric oxide binding proteins. Another postdoc (Holly Trueman) in the lab was making silk films. We used to joke that her silk films were so flexible we could use them as cling wrap to bring our sandwiches to work. Not only were the films flexible, they were also optically transparent and seemed perfect for use in an optical biosensor.

To begin with, we used silks as a way to immobilise heme proteins such as myoglobin. The active site of myoglobin is an iron porphyrin macrocycle, to which gases bind and give these proteins their distinctive colouration. The immobilised myoglobin was still able to reversibly bind nitric oxide. The myoglobin-silk films were remarkably stable at ambient conditions for months and were able to bind nitric oxide directly from the gaseous phase rather than us having to measure dissolved nitric oxide [7].

This success got us wondering if there was more we could do with silks. The silk protein we were using originated from honeybees. Tara Sutherland’s group at CSIRO has had remarkable success producing this silk in bacterial fermentation systems at commercially viable levels. The principle advantage of using honeybee silks over other recombinant structural proteins, is that these honeybee proteins tolerate significant modification of the protein sequence without compromising its ability to form stable materials, thereby allowing us to redesign the protein to introduce new functions [8,9].

Honeybee silk has a very different structure from silkworm silk. It is a coiled-coil protein containing ample alpha helices, similar to the tertiary structure of myoglobin and hemoglobin. In recent years there have been a number of success stories on the use of small peptides, which assemble into alpha helical bundles, to design man-made heme proteins; this field is known as de novo (from scratch) engineering. For example, Dutton and co-workers produced an iron porphyrin-peptide complex which has the same oxygen binding properties as hemoglobin [10].

By immobilising myoglobin in honeybee silk we were inserting an entire heme protein into the silk protein. Did we really need the globin protein backbone or could we replace the globin with the silk? This would leave us with the heme porphyrin ring wrapped in a silken coat. By immobilising the functional part of myoglobin in silk, could we produce a biomimetic myoglobin within a solid-state material, such as a sponge (Figure 2)?

Our first goal was to use the silk protein environment to control the reactivity of the metal centre through direct coordination of the silk to the iron centre, akin to that used in myoglobin (Figure 2). Using residues such as histidine within the hydrophobic core of the coiled-coil silk we obtained direct coordination of the residue to the iron centre and have been able to produce sensing heme-silk films (Figure 1B) [11].

Using these films, we were able to detect nitric oxide that bound to the iron heme centre as it resulted in a change in the UV-Vis spectrum. The heme-silk film showed an improved limit of detection for dissolved nitric oxide (1 µM) compared to myoglobin-silk films (5 µM) [7] and myoglobin in solution (10 µM) [12]. Given that the films are well suited to detecting nitric oxide in the gaseous phase, we are testing if our heme-silk films can be used as a diagnostic for the early detection of asthma through monitoring changes in the nitric oxide concentrations in exhaled breath. Nitric oxide is a good biomarker of lung inflammation in asthmatics and the stability of the heme-silk films lends itself to use in a portable breath analyser for use at home.

We have also been able to produce heme-silk sponges (Figure 1C). We demonstrated that these sponges have heme-peroxidase activity and can function as a recoverable heterogenous biocatalyst. The robustness of these new biomimetic materials is quite remarkable, as they maintain their functional properties after being stored, dry, at room temperature for over one year and they are stable in a range of organic solvents, weak acids, and bases [11]. This wide range stability allows heme-silk materials to be used in environments often not tolerated by naturally occurring proteins.

We are now varying the silk protein sequence to produce mimics of other heme proteins. For example, we are using cysteine rather than histidine coordinating residues, akin to the remarkably versatile family of cytochrome P450 enzymes. These enzymes are of significant interest in pharmaceutical applications.

Nature also uses a range of non-heme metal macrocycles in proteins, such as magnesium chlorophylls in photosynthesis. Inspired by these systems, we have used different metals (e.g., cobalt, zinc, and ruthenium) and different organic rings to develop silk materials with new functional properties. Using this approach we have developed silk films which generate cytotoxic oxygen species that could have applications as antimicrobial wound dressings [13].

Interestingly, our journey from using silk proteins for immobilisation to the development of an engineering scaffold for biomimetic materials is similar to that followed by a different class of materials, metal-organic frameworks (MOFs). For decades, metal-organic materials have attracted much research interest due to their tuneable pores. Proteins such as microperoxidase, cytochrome *c*, and myoglobin have been entrapped within MOFs to improve their stability. More recently, attention has shifted towards biomimicry by incorporating metalloporphyrins and iron-sulfur clusters into the MOF framework to produce biomimetic catalysts and sensors [14].

Although in its infancy, the field of biomimetic solid-state materials is proving to be a promising alternative to enzyme immobilisation. In particular, beginning with a stable material, such as a silk or an MOF, leads to a remarkably robust material with which to harness the diverse chemistry of natures’ metalloenzymes. Through varying the metal centre, organic cofactor, the protein sequence, and material format, we envisage the production of a range of solid-state materials with different functional properties, such as biosensors, biocatalysts, light harvesting materials, and therapeutic agents.

## Figures and Tables

**Figure 1:**
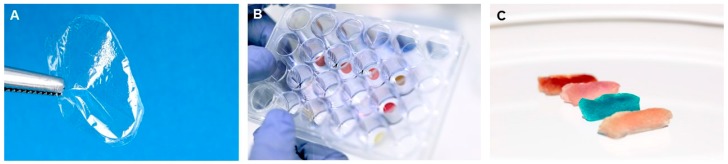
Photographs of solid-state silk materials. (**A**) Unmodified of recombinant honeybee silk fabricated into a film; (**B**) Honeybee silk films with various macrocycles cast in a 24-well plate; (**C**) Honeybee silk sponges with incorporation of heme *b* (red), cobalt corrin (pink), zinc phthalocyanine (blue) and palladium porphyrin (orange).

**Figure 2:**
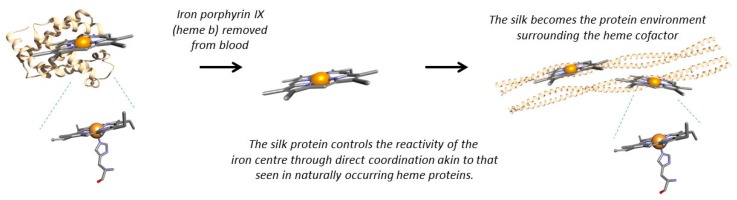
Scheme demonstrating how the silk protein replaces the heme protein scaffold to produce a silk based metalloprotein. The silk protein not only immobilises the heme cofactor but controls the reactivity of the metal centre via direct coordination of a histidine residue.
